# Pharmacological treatment of inhalation injury after nuclear or radiological incidents: The Chinese and German approach

**DOI:** 10.1186/s40779-019-0200-2

**Published:** 2019-03-31

**Authors:** Tian-Tian Yan, Guo-An Lin, Min-Jie Wang, Andreas Lamkowski, Matthias Port, Alexis Rump

**Affiliations:** 1 Military Burn Center, the 990th Hospital of the Joint Logistics Support Forces of Chinese PLA (the 159th Hospital of Chinese PLA), Zhumadian, 463000 Henan China; 2 Bundeswehr Institute of Radiobiology, Munich, Germany

**Keywords:** Fire smoke, Inhalation injury, Carbon monoxide, Cyanide, Radionuclide incorporation, Decorporation

## Abstract

Inhalation injury is often associated with burns and significantly increases morbidity and mortality. The main toxic components of fire smoke are carbon monoxide, hydrogen cyanide, and irritants. In the case of an incident at a nuclear power plant or recycling facility associated with fire, smoke may also contain radioactive material. Medical treatments may vary in different countries, and in this paper, we discuss the similarities and differences in the treatments between China and Germany. Carbon monoxide poisoning is treated by 100% oxygen administration and, if available, hyperbaric oxygenation in China as well as in Germany. In addition, antidotes binding the cyanide ions and relieving the respiratory chain are important. Methemoglobin-forming agents (e.g., nitrites, dimethylaminophenol) or hydroxocobalamin (Vitamin B12) are options. The metabolic elimination of cyanide may be enhanced by sodium thiosulfate. In China, sodium nitrite with sodium thiosulfate is the most common combination. The use of dimethylaminophenol instead of sodium nitrite is typical for Germany, and hydroxocobalamin is considered the antidote of choice if available in cases of cyanide intoxications by fire smoke inhalation as it does not further reduce oxygen transport capacity. Systematic prophylactic use of corticosteroids to prevent toxic pulmonary edema is not recommended in China or Germany. Stable iodine is indicated in the case of radioiodine exposure and must be administered within several hours to be effective. The decorporation of metal radionuclides is possible with Ca (DTPA) or Prussian blue that should be given as soon as possible. These medications are used in both countries, but it seems that Ca (DTPA) is administered at lower dosages in China. Although the details of the treatment of inhalation injury and radionuclide(s) decorporation may vary, the general therapeutic strategy is very similar in China and Germany.

## Background

In military conflicts, burns are expected to occur in 10–30% of the wounded depending on the scenario [1, 2]. Explosions, for example, from improvised explosive devices (IEDs) may lead to thermal injury directly by the heat released or indirectly by the ignition of combustible materials. It has been reported that on average, combat burns are associated with a higher injury severity score and a higher incidence of inhalation injury than noncombat burns [3, 4]. Although it has never happened previously, malicious contamination of IEDs with radioactive materials used in industry, research or medicine must be considered as a possibility (“dirty bomb” scenario). Such a scenario associating an explosion, fire and the liberation of radioactivity may result in a combined radiation injury (CRI) causing external irradiation, blast injuries, thermal and possibly radiation burns, as well as an inhalation injury from fire smoke associated with the incorporation of radionuclide(s) [5]. Additionally, in the case of nuclear detonation, a large number of victims with CRI (combined radiation injury-blast injuries, thermal burns + irradiation) are expected.

In addition to military settings, occupational accidents in industrial plants may result in a large number of burn patients [6, 7]. Because of the frequent storage of various chemicals, the associated inhalation injuries caused by fumes in industrial fires may be particularly severe [8]. This may also happen during military operations when facilities with stored dangerous materials are damaged in combat inadvertently or voluntarily [9]. In German burn centers, on average, inhalation injury complicates approximately 20 - 30% of cases [2, 10, 11]. In nuclear power plants, fire is also a major hazard and may occur in critical locations with a risk of fuel damage [12]. In February 2017, for example, an explosion and fire occurred in the turbine room of the nuclear plant in Flamanville in northwestern France, and several workers had to be evaluated for smoke inhalation [13]. Although in this case the accident happened outside the “nuclear zone” and there was no radioactive contamination, the liberation of radioactivity is a possibility that must be considered. In autumn 2017, low levels of the radionuclide ruthenium-106 were detected in the atmosphere in different European countries [14]. The source was traced back to the south of the Ural Mountains. As ruthenium-106 was found in isolation and not in combination with other radionuclides, a leak from a reprocessing plant separating ruthenium was suspected rather than a power plant accident. Considering the physicochemical properties of the metal ruthenium, it was concluded that the accident must have been accompanied by a massive heat development (temperatures of 800–1000 °C), probably from a fire causing vaporization of ruthenium and its penetration into the higher air layers of the atmosphere [15]. Although no precise information is available in this particular case, this kind of accident is suited to cause a combination of thermal and inhalation injuries, as well as external irradiation and radionuclide incorporation (“thermal CRI”) among personnel working at the facility.

The objective of this paper is not to discuss all aspects of thermal CRI but to briefly review the toxic properties of fire smoke, taking into account additional radioactive contamination and inhalation of radionuclide(s), with a focus on pharmacological treatment options. In particular, the similarities and differences in the therapeutic approaches in China and Germany are highlighted. For this purpose, a selective literature search was conducted that included papers written in English, German and Chinese languages.

Carbon monoxide and hydrogen cyanide as components of fire smoke may result in a very short-term occurrence of death due to systemic toxicity. Airway injuries are the consequences of the thermal effects of heated gases and direct chemical injury of the airways by irritants and will manifest within hours to days. The inhalation of radionuclides leading to internal contamination will most likely result only in long-term stochastic health effects. The different agents and effects are discussed according to the timelines of the clinical manifestations they induce.

### The systemic toxicity of fire smoke components

#### Carbon monoxide

Carbon monoxide is generated by incomplete oxidation of carbon and must always be expected in fire smoke. It may be suspected from symptoms ranging from headache to coma, but clinical diagnosis is uncertain as the cherry red skin coloration described in textbooks is seldom seen in real clinical settings [16]. Mild carbon monoxide intoxication may sometimes be confused with psychiatric syndromes [17]. In the inspired air, 2% carbon monoxide is enough to transform 60% of hemoglobin into carboxyhemoglobin within approximately 2 min [18, 19]. This level is usually considered lethal. Oxygen saturation readings do not permit the diagnosis of carbon monoxide intoxication, and normal partial arterial oxygen pressure (PaO_2_) does not permit the exclusion of poisoning. For this purpose, particular oximeters measuring carboxyhemoglobin must be used (e.g., RAD-57 Oximeter, Masimo).

Carbon monoxide binds to hemoglobin with an affinity 200 to 250 times greater than oxygen. The oxygen transport capacity of the blood is reduced, leading to hypoxia. Moreover, the hemoglobin dissociation curve is shifted to the left, further compromising the delivery of oxygen to the tissues [17]. In mitochondria, carbon monoxide also binds to cytochrome oxidases, interfering with cellular respiration [20]. In addition to neurologic symptoms, vasodilation, a decrease in cardiac output and/or acute coronary syndromes have been described. ECG disturbances and elevated biomarkers of myocardial tissue damage are frequent in patients poisoned with carbon monoxide [17, 21, 22]. The symptoms depend on the amount of carboxyhemoglobin formed and the remaining hemoglobin available for oxygen transportation (Table 1) [23]. However, there is only a loose relation between carboxyhemoglobin levels and symptoms, and the whole clinical situation should be taken into account when assessing the patient [22]. Carbon monoxide poisoning in fire victims is associated with higher mortality and may result in lasting neurologic sequelae and myocardial injury [24].

**Table 1:** Symptoms of carbon monoxide poisoning depending on the level of carboxyhemoglobin in the blood

Carboxyhemoglobin (%)	Symptom
< 5	No symptoms
5–10	Visual impairments
10–20	Headaches, palpitations, dyspnea
20–30	Worsening of symptoms
30–40	Somnolence, unconsciousness
40–60	Coma
> 60	Death

In addition to carboxyhemoglobin levels, symptoms also depend on the absolute hemoglobin concentration and the health status of the victim. Source: [23]

**Fig. 1:**
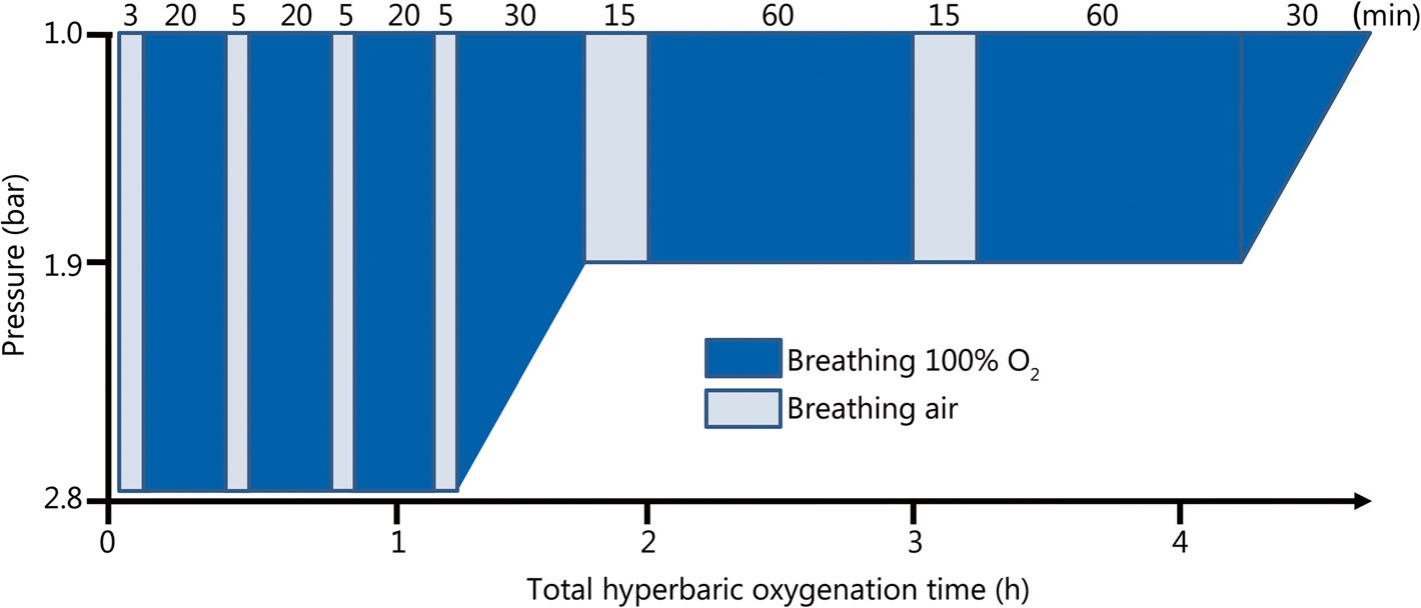
US Navy hyperbaric oxygenation treatment Table 6 (USN TT6, “standard of care”, which is also used to treat acute carbon monoxide intoxications; many variants exist). One bar absolute pressure is the usual ambient pressure and corresponds to a 10 m column sea water pressure, i.e., at the surface, the absolute pressure is 1 bar and at a 10 m depth, the pressure is 2 bar. One hundred percent oxygen is alternated with short air-breathing periods (time intervals provided in columns) to reduce acute oxygen toxicity at high pressure manifesting in the central nervous system (e.g., convulsions)

The treatment of carbon monoxide poisoning is by administering 100% oxygen (e.g., in spontaneously breathing patients via a mask with a reservoir and high oxygen flow) to displace carbon monoxide from hemoglobin, speeding up its pulmonary elimination and improving the oxygen transport capacity of the blood [22, 25]. High oxygen partial pressures, as in hyperbaric oxygenation (HBO), can be used to further enhance the elimination of carbon monoxide (half-time of carbon monoxide when breathing ambient air (21% oxygen): 4–5 h; breathing 100% oxygen: approximately 1.0–1.5 h; hyperbaric oxygenation at 2.8 bar (0.28 MPa, corresponding to a diving depth of 18 m sea water) absolute pressure and breathing 100% oxygen: 15–30 min) [16, 22, 26]. Carbon monoxide is considered an indication for applying US Navy Table 6 (“standard of care”) as a treatment scheme for the first session (Fig. 1), but the selected overpressure may vary depending on clinical conditions and the habit of the hyperbaric center and is usually reported between 2.5 and 3.0 bar (0.25–0.30 MPa) absolute pressure [22, 27, 28]. It seems that carbon monoxide bound to hemoproteins in the tissues has a longer elimination half-time that may reach 48 to 72 h when breathing ambient air [17]. In addition to rapidly improving oxygen transport capacity, it was reported that cognitive sequelae in carbon monoxide poisoning might be reduced by repetitive hyperbaric oxygenation treatment sessions, which was explained by more rapid restoration of cytochrome oxidase activity in the brain [29]. According to the European Committee for Hyperbaric Medicine (Consensus Conference 2004), there is an indication for hyperbaric oxygenation treatment in unconscious patients or those showing neurologic, cardiac, respiratory or psychological symptoms [30]. An indication for hyperbaric oxygenation in the case of carbon monoxide poisoning is also seen in pregnant women, as the invasion and elimination of carbon monoxide seems to be slower in fetal blood, explaining why carboxyhemoglobin levels may still be high in the fetus when time values have already decreased and are noncritical in maternal blood [22]. However, there is no conclusive evidence that hyperbaric oxygenation actually improves the clinical outcome after carbon monoxide intoxication [22, 24, 25]. Moreover, the number of hyperbaric chambers ready to admit patients anytime on short notice is very limited, and only a few are equipped and staffed to manage intensive care patients. Thus, in many cases, the specific therapy of carbon monoxide poisoning is limited to the administration or ventilation with 100% oxygen at ambient pressure.

In China, the inhalation of 100% oxygen is also widely accepted as an effective treatment for carbon monoxide poisoning, and the application of hyperbaric oxygen therapy is also considered highly necessary [31, 32]. By enhancing the early and quick discharge of carbon monoxide, hyperbaric oxygen therapy helps the patient recover consciousness and reduces adverse consequences of anoxia. In China, the goal is to treat patients with carbon monoxide poisoning in hospitals with hyperbaric chambers as early as possible in the acute phase. The treatment normally lasts for 60 min at a pressure of 2 to 2.5 bar (0.20–0.25 MPa). Treatment sessions may be repeated depending on the patient’s condition. Hyperbaric oxygen therapy is usually applied only when the patient is in stable condition and patients are accompanied in the chamber by an experienced doctor or nurse. According to the results observed in China, the positive effect of hyperbaric oxygen therapy in carbon monoxide poisoning is very significant. A retrospective study showed that hyperbaric oxygenation significantly decreased patients’ risk of mortality [33]. According to the national guidelines on carbon monoxide poisoning treatment published by the Chinese Association of Hyperbaric Medicine, its use is recommended as soon as possible after intoxication, provided the practitioners and staff are conscious of and able to deal with severe medical conditions (e.g., pulmonary edema, heart failure) in the particular environment of a hyperbaric chamber [34–36]. There is also a scientific interest in the possibility of combining hyperbaric oxygenation with the use of acupuncture or Chinese medicine, which is not utilized in Germany, to further improve neurological outcomes [37–39]. Generally, under hyperbaric oxygenation, potential interactions between nonphysiologically high oxygen partial pressures and drug effects (Western or Chinese) must be taken into account [40].

#### Hydrogen cyanide

Hydrogen cyanide may be formed during the combustion of nitrogen-containing natural or synthetic materials (e.g., wool, nylon or polyurethane). The hydrogen cyanide concentration in combustion gases is temperature dependent, and it has been shown that toxicologically relevant hydrogen cyanide concentrations can be present at the beginning of the fire but subsequently drop to low levels [41]. Thus, if hydrogen cyanide is not measurable in the atmosphere, this does not necessarily exclude hydrogen cyanide intoxication in burn victims.

**Fig. 2:**
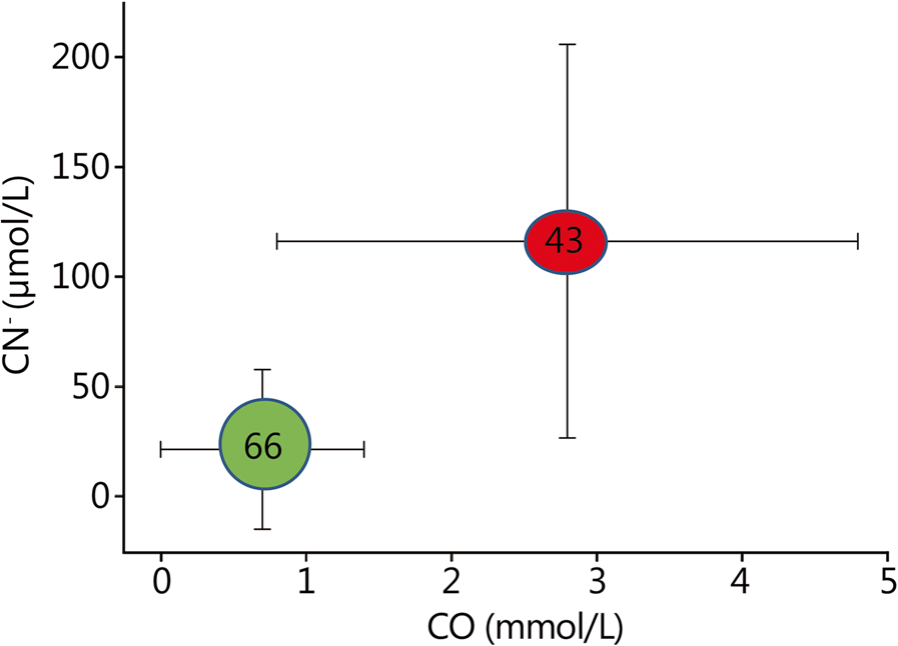
Cyanide and carbon monoxide concentrations in the blood of victims of residential fires classified by outcome, with 66 survivors (green color) and 43 fatalities (red color) (mean ± standard deviation). Source of the data: [43]. A total of 2.7 mmol/l carbon monoxide in the blood roughly corresponds to 30% carboxyhemoglobin [17]

The real significance of hydrogen cyanide poisonings in fire victims has been controversially discussed [24, 42]. From the analysis of blood samples obtained during autopsies of burn victims, it has been concluded that cyanide intoxications are relatively rare and usually associated with very high levels of carboxyhemoglobin [42]. However, the half-life for cyanide in humans was determined to be approximately 1 h [43], so concentrations determined from the autopsy in victims who possibly survived several hours before death or patient blood sampled late after rescue may lead to erroneous conclusions. A clinical investigation with very early blood sampling for cyanide determination at the scene of the fire showed that non-surviving burn victims had higher blood concentrations of carbon monoxide and cyanide (Fig. 2) [43]. There was no significant correlation between the extent of the burn wounds and the cyanide or carbon monoxide concentrations. Among the non-surviving burn victims, 74% showed toxic (> 40 μmol/L, 1 mg/L) and 46% showed potentially lethal (> 100 μmol/L, 2.6 mg/L) cyanide concentrations [43]. Cyanide blood concentrations in the range of 0.5–1.0 mg/L cause mild symptoms (headache, dizziness, confusion, poor vision, slurred speech), 1–2 mg/L causes moderate (dizziness, chest pain, palpitations, arrhythmias) and 2–3 mg/L causes severe symptoms (unconsciousness, cardiovascular collapse, convulsions, respiratory arrest) [44, 45]. Concentrations over approximately 3 mg/L are usually considered lethal [46]. There is only a very limited correlation between the carbon monoxide and cyanide concentrations, so carboxyhemoglobin levels should not be used to exclude or to predict the severity of cyanide intoxication [46]. These findings show that the possibility of cyanide intoxication must always be considered in burn victims.

Although a rapid diagnosis is necessary for critical patients, there is no quick and reliable test to measure cyanide concentrations in biological material at the point of care, and clinical signs are not specific, particularly in the presence of additional carbon monoxide intoxication. Elevated lactate levels may be used as one indication of a possible cyanide poisoning [24, 47]. Thus, treatment decisions are based on a probabilistic evaluation of all information available about the scene and the patient.

Cyanide ions have a high affinity for trivalent (not divalent) iron and bind to cytochrome oxidases, blocking the mitochondrial respiratory chain and leading to inner suffocation. In addition to cytochrome oxidase, numerous other metal enzymes are also inhibited. By coupling to sulfur, cyanide is rapidly detoxified (approximately 0.1 mg/ (kg·h) in humans to 1 mg/ (kg·h) in animal studies) to the far less toxic thiocyanate (= rhodanide) that is largely excreted unchanged through the kidneys. The factor limiting the detoxification of cyanide is the availability of sulfur from intermediary metabolism [48].

Physicians in China and Germany deal with cyanide poisoning using the same therapeutic principles. Nevertheless, there are differences among the drugs approved and available (Table 2). The treatment of cyanide intoxication must include the administration of oxygen, as even with exposure to lethal concentrations, the residual cyanide-insensitive activity of cytochrome oxidases seems to remain [47]. Nevertheless, there is a clear indication for the use of antidotes with the goal of relieving the respiratory chain and speeding up cyanide metabolism [47, 48]. The elimination of cyanide can be enhanced by sodium thiosulfate (0.10–0.25 g/kg body weight, *i.v*.), increasing the availability of sulfur. However, the therapeutic effect is delayed, and in severe life-threatening situations, the administration of thiosulfate alone is insufficient. In severe poisonings, agents that induce the formation of methemoglobin (hemoglobin with trivalent instead of divalent iron) may be used, as the trivalent iron of methemoglobin binds cyanide ions, thus, the cytochrome oxidases will be relieved. For this purpose, amyl nitrite or sodium nitrite may be used [16]. Amyl nitrite inhalation treatment has been known for a long time and may be useful as a first aid measure, but the amount of generated methemoglobin may be quite low (< 6%) [49]. Sodium nitrite as a cyanide antidote is probably used most commonly internationally, but the treatment may be associated with severe hypotension [50]. In addition to methemoglobin formation, it has been suggested that the antidotal activity of sodium nitrite might also be mediated by the generation of nitric oxide that displaces cyanide from cytochrome oxidase [51]. In Germany, dimethylaminophenol (DMAP) is preferred over sodium nitrite as its hypotensive effect is less pronounced, and at the recommended dosages, the formation of methemoglobin is faster and reaches higher concentrations (DMAP 3–4 mg/kg body weight leads to 30 - 35%). The downside is that methemoglobin cannot bind oxygen, and in the presence of carbon monoxide intoxication, the oxygen transport capacity of the blood may decrease to a critical life-threatening level. Therefore, the use of methemoglobin-forming agents in fire smoke victims is considered contraindicated by some authors [11, 25], whereas others accept its use in vitally endangered patients with a strong suspicion of cyanide poisoning, in part recommending a lower DMAP dosage (1–2 mg/kg). A better alternative that should be utilized if available is the administration of hydroxocobalamin (vitamin B12), which directly binds cyanide and thus relieves cytochrome oxidases (recommended dosage 70 mg/kg body weight) (1 pack Cyanokit® contains 2 vials of 2.5 g or 1 vial of 5.0 g hydroxocobalamin as a freeze-dried substance) [11, 16, 25, 48, 52, 53]. The starting dose for adults is 5 g intravenously. The formed cyanocobalamin is then excreted through the kidneys. The antidote is usually well tolerated even in the high doses required, and the difference to methemoglobin forming agents is that it does not interfere with oxygen transport in the blood. Dicobalt edetate (Kelocyanor), which contains cobalt, can be used as another treatment compound to bind cyanide, but serious cardiovascular side effects have been reported. These adverse effects are particularly pronounced when there is no cyanide intoxication, which is why this antidote should be administered only if the diagnosis is certain [45].

**Table 2:** Antidotes mostly used in China and Germany against toxic constituents of fire smoke

Poison	Antidotes	
	China	Germany
Carbon monoxide	100% Oxygen [31, 32] Hyperbaric oxygenation [31, 32]	100% Oxygen [22] Hyperbaric oxygenation [22]
Cyanide	Sodium nitrite [54, 55] Sodium thiosulfate [54, 55]	Dimethylaminophenol [48] Hydroxocobalamin [25, 48] Sodium thiosulfate [48]
Radionuclide(s)	Stable iodine [77] Ca(DTPA)* [77, 78] Prussian Blue [77]	Stable iodine [71] Ca(DTPA) [71] Prussian Blue [71]

Most differences in the drugs used concern cyanide poisoning treatment. *Ca(DTPA) has been reported to be used at lower dosages in China [78]

In China, a combination of sodium nitrite and sodium thiosulfate is the standard for the treatment of cyanide intoxication [54, 55]. DMAP is known in Chinese literature and is sometimes reported to be fast and efficient. However, it is not used as there is no approval for this drug [54]. The use of hydroxocobalamin as an antidote has not yet been reported in China [55].

#### Local injury of the airway

Thermal airway injury is usually limited to the supraglottic structures because of heat dissipation in the upper airway [19, 24]. Pharyngeal and laryngeal edema may develop rapidly, and the suspicion of upper airway obstruction should lead to early intubation or a tracheostomy [16, 24]. Edema and obstruction usually peak at 24 h and improve afterwards. Different from heated gas, the inhalation of steam has a much higher heat-carrying capacity than dry heat and may also affect the lower airway [16]. In addition to thermal injury, fire smoke may contain irritants causing chemical injuries to the subglottic region down to the bronchioles and alveoli (for example, hydrogen chloride as a combustion product from polyvinyl chloride, nitrous gases, sulfur dioxide, aldehydes such as acrolein, etc.). Various combustion products may be produced with differing penetration capacities depending mainly on solubility but also on their concentration. By dissolving in water, these products form corrosive acids or alkalis that will damage the cell lining of the airway. Moreover, particulate matter may be inhaled. The respiratory mucosa and possibly the alveolar wall may be injured, leading to an inflammatory response. The mechanism is very complex and involves the stimulation of vasomotor and sensory nerve endings, the release of neuropeptides and the induction of neutrophil chemotaxis [24]. The generation of reactive oxygen species (ROS), such as superoxide anions (O_2_^−^), hydroxyl radicals (OH^−^) or hydrogen peroxide (H_2_O_2_), is also involved in these pathophysiological mechanisms. Free radicals are chemically highly reactive and short-lived entities are damaging all kinds of cellular structures. At the functional level, the loss of plasma from the intravascular compartment into the alveoli and bronchioles combined with bronchospasms will impair gas exchange and cause hypoxia. Airway obstruction and atelectasis may occur. The loss of hypoxic pulmonary vasoconstriction will lead to an increase of blood flow to poorly ventilated areas, further deteriorating blood oxygenation by a ventilation/perfusion mismatch. Moreover, bacterial clearance will be reduced, enhancing the risk of pneumonia [19].

In addition to the clinical assessment of the situation at the site of the incident, several techniques may be used to assess airway injury. In Germany and China, fiberoptic bronchoscopy is still the standard to assess the severity of the lesions and permits pulmonary hygiene to be performed [2, 56, 57]. Bronchial lavage can also remove sticky secretions, necrotic tissue and carbon particles deposited in the trachea and lungs. However, it should not be performed repeatedly without a particular indication (e.g., atelectasis) as it causes irritation and injury of the mucous membrane of the airway [2]. Additional complications, such as the occurrence of bronchospasms, hemorrhage or pneumothorax, must also be considered [58]. Moreover, at the time of the initial assessment, this technique does not permit visualization of the distal airway. Chest computed tomography may also be used by applying different score systems [24]. Respiratory function is assessed by blood gas analysis and the PaO_2_/FiO_2_ ratio and may be used as an indicator for the severity of inhalation injury. In addition to respiratory support by the administration of oxygen and/or mechanical ventilation using different modes, bronchodilators (e.g., salbutamol, nebulized epinephrine) can be used to decrease airflow resistance and to improve dynamic compliance and the PO_2_/FiO_2_ ratio. By relieving bronchoconstriction, reducing secretions and, in the case of epinephrine, vasoconstriction, the ventilation/perfusion mismatch is improved. In addition, it seems that beta agonists and muscarinic receptor antagonists also exert anti-inflammatory activities. Mucolytic agents may be used to break up airway secretions. In particular, N-acetylcysteine is thought to have an additional beneficial effect by its antioxidative radical scavenging properties.

A difficult question arises regarding the use of glucocorticoids for the prophylaxis of toxic pulmonary edema. The earliest possible administration of dexamethasone isonicotinate through inhalation (Auxiloson®) was promoted for a long time in Germany and was still considered as a requirement in the 1980s. In the 1990s, the equivalence or even possible superiority of inhaled budesonide (Pulmicort®) for this indication was frequently explored [23]. In contrast to dexamethasone nicotinate, budesonide is not a prodrug and has a higher affinity for glucocorticoid receptors in human lung tissue. Several beclomethasone dipropionate inhalation preparations received official approval by the German Federal Drug Agency (BfArm) in 2000 for the prevention of toxic pulmonary edema after smoke inhalation (e.g., Junik® Autohaler, Ventolair® metered aerosol) [59, 60]. However, despite positive effects in animal experiments and indications of their possible usefulness after the inhalation of irritants, the efficacy of glucocorticoids to prevent toxic pulmonary edema has never been confirmed. In advanced hazmat life support (AHLS) protocols, glucocorticoids are not recommended after irritative gas inhalation [61]. The bulk of the literature in Europe and the US state that the prophylactic use of corticosteroids to prevent pulmonary edema is critical as the risk of infection is increased, and negative outcomes associated with their use in burn patients have been reported [2, 62–64]. However, corticosteroids may be indicated in burn patients with inhalation injury to treat severe bronchospasms, and in this case, an intravenous administration should be preferred [2, 11]. In China, although steroids are not recommended systematically for the prophylaxis of toxic pulmonary edema, dexamethasone sodium phosphate injections are also used to reduce laryngeal edema caused by inhalation injury. In the past, it was often inhaled atomically; however, with some patients suffering serious side effects, it is now highly recommended to be used intravenously.

Systemic inflammatory response syndrome (SIRS) is a common response to severe inhalation injury, infection and burn. The plasma concentration of inflammatory mediators in patients is significantly increased, and vascular endothelial cells and neutrophils are activated, leading to the accumulation of neutrophils in important organs in the body. SIRS may lead to multiple organ dysfunction syndrome (MODS), which is an important cause of death in patients with inhalation injury. Timely prevention of SIRS is critical for the success of the treatment.

Ulinastatin is a glycoprotein with broad-spectrum protease inhibition that is currently approved in China, India, Japan, and South Korea for a variety of indications [65]. It is widely used in clinical treatments in China at the dosage of 10–200,000 units every 8 h for 5–7 days, although there is still no standard guideline. It can significantly improve the indicators of systemic inflammatory response and control the development of SIRS and MODS through inhibiting the activity of a variety of proteolytic enzymes and regulating the release of inflammatory cytokines, and it has been shown to reduce mortality in acute burn patients [65].

#### The inhalation of radionuclides

In the case of an accident in a nuclear facility, the release of radioactive materials, such as radioiodine, actinides, and radioactive isotopes of cesium, must be considered. Radioactive materials that might be used in a “dirty bomb” attack cannot be predicted with certainty, but the radionuclide(s) widely used in industry, medicine or research must be considered due to their availability [66, 67]. Thus, fire smoke, gases, and dust may be radioactively contaminated and lead to the incorporation of radionuclides through inhalation. Past experiences indicate that the incorporation of radionuclides usually does not lead to acute radiation sickness [68], exceptions notwithstanding (e.g., the Litvinenko case) [69]. In the case of high exposures, however, radiation pneumonitis followed by fibrosis must be considered as a possibility [70]. Effects of the combination of thermal, chemically corrosive, and radiation on the inflammatory response of the airway are still unclear. Bronchial lavage has been advocated, particularly in cases of inhalation of relatively insoluble radioactive materials and high exposures [70]. In practice, lavage seems to be performed extremely rarely after radionuclide inhalation. In the case of thermal CRI patients with inhalation injury having fiberoptic bronchoscopy for diagnostic reasons, the opportunity should be used for pulmonary hygiene, removing obstructing secretions and at the same time reducing the radioactive contamination of the airway.

Except in the case of very high radioactive exposures, acute radiation sickness is usually not expected after radionuclide inhalation [68]. However, the incorporation and internal contamination with radionuclides deposited in organs and tissues may cause long-lasting internal irradiation with health effects, such as cancer development, manifesting many years later. The committed effective dose absorbed may be reduced by the administration of blocking agents and decorporation agents, the most important being stable iodine, DTPA and Prussian blue [71, 72]. In case of a radiological emergency, these antidotes are included in several national stockpiles.

After the inhalation or ingestion of radioiodine, its absorption into the thyroid may be competitively inhibited by the administration of stable iodine (100 mg iodine, i.e., 130 mg of potassium iodine per os in adults), substantially reducing the radiological dose absorbed by the gland and the probability of developing a thyroid carcinoma [73]. However, the time of administration is crucial. Stable iodine is most effective if given less than 12 h prior or up to 2 h after radioiodine exposure [74]. It is less effective thereafter. This view is internationally acknowledged [71]. It should not be administered later than 24 h after radioiodine exposure, as this may even be deleterious by extending the presence of radioiodine that has already been taken up into the thyroid. In a short-term exposure, a single dose of stable iodine is sufficient. Repetitive doses should only be considered in the case of prolonged radioiodine exposures that cannot be avoided.

(Ca)DTPA (diethylenetriaminepentaacetic acid) (1 g / day, *i.v.*) can bind many transuranic metals (e.g., plutonium, americium, curium) or rare earth (e.g., cerium) and enhance their renal excretion, thus reducing the committed effective doses [71, 75]. Prussian blue (usually 3 × 1 g orally) can bind cesium-137, which is, in part, secreted through the bile into the lumen of the intestine, preventing its reabsorption from the gut and thus enhancing its elimination through the feces [76]. Both drugs are available and recommended in Germany [71] as well as in China [77], but it seems that there are differences in the dosages for Ca (DTPA). Whereas 1 g/day is recommended in Europe, it is reported that in China, the initial dose is lower (0.1–0.5 g) and further reduced to 0.1 g/day in an extended course [78].

**Fig. 3:**
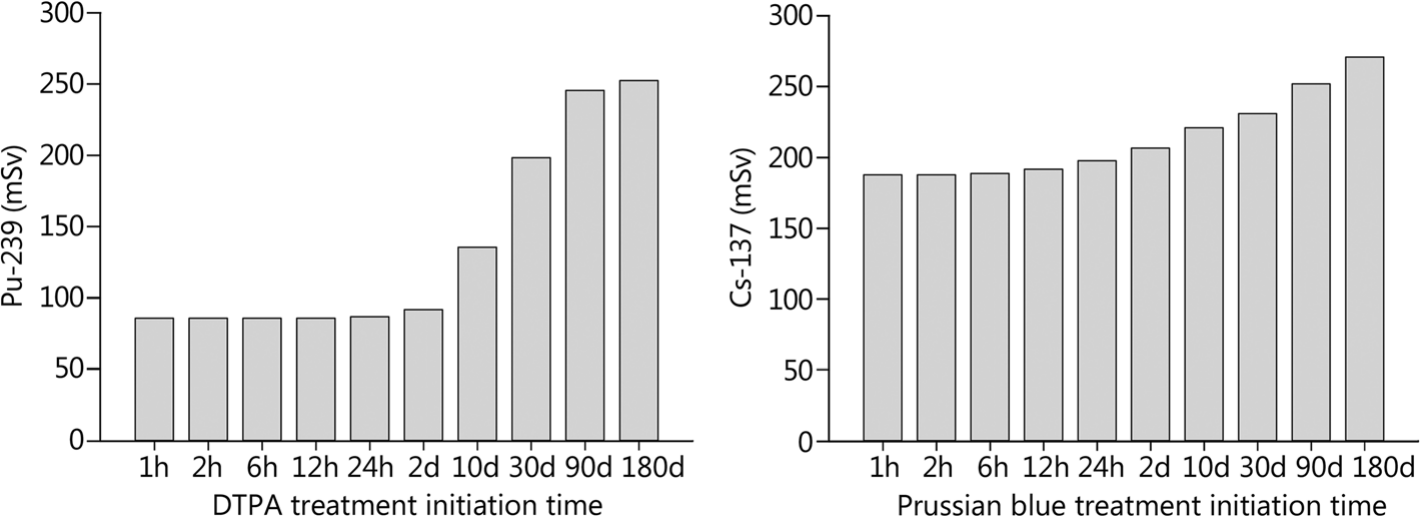
Impact of the time of initiation of decorporation treatment on the committed effective doses (mSv) after the inhalation of 1 μCi of Pu-239 as a poorly soluble compound (for example, PuO_2_) or 1 mCi of Cs-137 as a soluble compound (for example, CsCl). A treatment duration of 30 days is assumed. The committed effective doses without treatment amount to 261 mSv for Pu-239 and 291 mSv for Cs-137. Source of the data: [79]

For decorporation treatments by chelating agents, there is still no consensus when to start treatment [71]. According to the “precautionary approach”, the results of internal dosimetry should be awaited, and only patients with relevant internal contamination should be treated (committed effective dose < 20 mSv: no treatment; 20–200 mSv: treatment should be considered; > 200 mSv: treatment should be implemented) [71, 75]. However, it may take days and even weeks before internal dosimetry results are available, and therapeutic efficacy decreases if treatment initiation is delayed (Fig. 3) [79]. This is due to pharmacokinetic reasons; radionuclides, such as plutonium, enter “deep compartments” (e.g., bone and liver tissue) where they cannot be bound by Ca (DTPA), which mostly distributes only in the extracellular space. According to the “urgent approach”, all victims with suspected incorporation of radionuclides should be given decorporation agents until relevant internal contamination has been excluded by measurement [80]. This is the treatment approach preferred by the Bundeswehr Institute of Radiobiology [71], although in the case of large-scale scenarios, it would cause a logistical challenge [81].

The urgency of decorporation is, however, relative and depends on the radionuclide(s), the invasion pathway (absorption by inhalation, ingestion or through a wound), the physicochemical properties of the material involved and, particularly, the solubility. It is not possible to recommend a clear cut time frame, but depending on the situation, therapeutic efficacy must be expected to decrease within several hours to several days [79, 82]. The bottom line is that even if acute health effects by the incorporation of radionuclides are not expected, treatment should be rapidly implemented after exposure to avoid or mitigate long-term health effects.

## Conclusion

An inhalation injury by fire smoke combined with exposure to radioactivity in a setting probably associated with further burns and/or mechanical trauma is certainly an uncommon injury pattern. However, that is why it is important to have a clear idea of the timelines of the pathophysiological processes involved. The basic rule applies to all emergencies: “treat first what kills first”. Triage and emergency treatment should follow the general principles of trauma care as established, for example, in the PHTLS (prehospital trauma life support) and ATLS (advanced trauma life support) concepts [16]. The first priority has always been the maintenance of vital functions, particularly good oxygenation and the treatment of carbon monoxide and hydrogen cyanide intoxications. Airway injury treatment must be adjusted to functional impairments. Although the deleterious health effects of radionuclide(s) incorporation will manifest only in the long term, it is important to remember that for a highly effective decorporation treatment, the “opportunity window” is limited in time.

In addition to the administration of specific drugs, comprehensive therapy, such as regulating the release of inflammatory cytokines, sedation, analgesia, tracheal intubation, and ventilator-assisted breathing, is crucial during the whole process. These methods help to reduce oxygen consumption, increase oxygen supply, protect vital organs and improve the condition of the body, thus reducing mortality.

Health systems, the organization of emergency systems and hospitals, as well as treatment procedures, including the use of particular medications and their recommended dosages, may differ from country to country. Although the details of the treatment of inhalation injuries and radionuclide(s) decorporation may vary, due to some differences in the drugs approved and available in both countries (Table 2), the general therapeutic strategy is nevertheless very similar in China and Germany.

## Abbreviations

AHLS: Advanced hazmat life support
ATLS: Advanced trauma life support
CRI: Combined radiation injury
DMAP: Dimethylaminophenol
DTPA: Diethylenetriaminepentaacetic acid
IED: Improvised explosive device
Iv: Intravenous
MODS: Multiple organ dysfunction syndrome
mSv: Millisievert
PHTLS: Prehospital trauma life support
ROS: Reactive oxygen species
SIRS: Systemic inflammatory response syndrome
